# Impact of gestational weight gain on obstetric and neonatal outcomes in obese diabetic women

**DOI:** 10.1186/s12884-015-0692-z

**Published:** 2015-10-08

**Authors:** Inês Gante, Njila Amaral, Jorge Dores, Maria C. Almeida

**Affiliations:** Department of Obstetrics, Maternidade Bissaya Barreto - Centro Hospitalar e Universitário de Coimbra, Rua Augusta, 3000-061 Coimbra, Portugal; Department of Obstetrics, Hospital Beatriz Ângelo, Loures, Portugal; Department of Obstetrics, Centro Hospitalar do Porto, Porto, Portugal

**Keywords:** Obesity, Gestational diabetes mellitus, Gestational weight gain, IOM recommendations, Pregnancy outcomes

## Abstract

**Background:**

Both obesity and gestational diabetes mellitus are increasing in prevalence, being a major health problem in pregnancy with independent and additive impact on obstetrics outcomes. It is recognized that inadequate gestational weight gain is an independent risk factor for pregnancy-related morbidity. The aim of this study was to evaluate the effect of gestational weight gain on obstetric and neonatal outcomes in obese women with gestational diabetes.

**Methods:**

Retrospective multicenter study of obese women with gestational diabetes. The assessed group was divided into three categories: women who gained below (<5 kg), within (5–9 kg) and above (>9 kg) the 2009 Institute of Medicine recommendations. Maternal and neonatal outcomes were compared and adjusted odds ratios calculated controlling for confounders.

**Results:**

Only 35,1 % of obese women with gestational diabetes (*n* = 634) achieved the recommended gestational weight gain; 27,8 % (*n* = 502) gained below and 37,1 % (*n* = 670) above the recommendations. There was a positive correlation between gestational weight gain and neonatal birthweight (*r* = 0,225; *p* < 0,001). Gestational weight gain below recommendations was associated with lower odds for cesarean section, even adjusting for birthweight [aOR = 0,67 (0,54–0,85); *p* < 0,001]; lower odds for large for gestational age neonates [aOR = 0,39 (0,28–0,57); *p* < 0,001] and macrosomia [aOR = 0,34 (0,21–0,55); *p* < 0,001]. Excessive weight gain, even adjusting for birthweight, was associated with higher odds for cesarean section [aOR = 1,31 (1,07–1,61); *p* = 0,009], low Apgar score [aOR = 4,79 (1,19–19,21); *p* = 0,027], large for gestational age neonates [aOR = 2,32 (1,76–3,04); *p* < 0,001] and macrosomia [aOR = 2,39 (1,68–3,38); *p* < 0,001].

**Conclusions:**

In obese women with gestational diabetes, a reduced gestational weight gain (<5 kg) is associated with better obstetric and neonatal outcomes than an excessive or even an adequate weight gain. Therefore, specific recommendations should be created since gestational weight gain could be a modifiable risk factor for adverse obstetric outcomes.

## Background

Both obesity and gestational diabetes mellitus (GDM) are increasing in prevalence, being a major health problem in pregnancy [1–4].

Obesity (defined as body mass index (BMI) ≥ 30 kg/m^2^) is a chronic illness which prepregnancy incidence is around 20 % [2–6]. It is an independent risk factor for obstetric complications, such as congenital abnormalities, GDM, preeclampsia, large for gestational age (LGA) and macrosomic newborns, fetal distress, low Apgar scores and cesarean sections [2, 4, 5, 7–10].

GDM is one of the most common medical complications of pregnancy [11]. The obstetric complications associated are well-known: preterm labor, preeclampsia, LGA and macrosomia, growth restriction, birth trauma, cesarean section, neonatal hypoglycemia, among others. [2, 4, 5, 7, 8, 11].

The impact of maternal obesity and GDM on obstetrics outcomes appears to be independent and additive [2, 4, 5, 10].

It is recognized that inadequate gestational weight gain (GWG) is an independent risk factor for pregnancy-related morbidity. Overall and in obese women, excessive early GWG is associated with adverse pregnancy outcomes including GDM, cesarean section and LGA [12–15]; insufficient GWG has been (although not consistently) associated with small for gestational age (SGA) newborns [13–16].

In 2009, the Institute of Medicine (IOM) updated guidelines for GWG, formulated as a range for each category of prepregnancy BMI. In obese women, the recommended range for GWG is 5–9 kg [17]. However, no coexisting pregnancy complications (such as GDM) were considered in these guidelines.

In women with GDM, recent data have shown that excessive GWG confers an additional risk for LGA, macrosomia, gestational hypertension, cesarean section and low Apgar scores [10, 18]. Therefore, prevention of excessive GWG has the potential to reduce these adverse obstetric outcomes [10].

Furthermore, there are some studies that suggest that a GWG below IOM recommendations in overweight and obese women with GDM could result in more favorable pregnancy outcomes without an increase in SGA risk [19, 20].

Considering these data, we hypothesized that in obese women with GDM, the intervention on GWG could have a relevant impact on obstetrics outcomes: excessive GWG confers an additional risk for adverse outcomes; insufficient GWG decreases the risk of adverse outcomes. In this study we will describe the effects of excessive, adequate and insufficient GWG (defined using IOM criteria) within a cohort of obese women diagnosed with GDM.

## Methods

A retrospective cohort study of Portuguese obese pregnant women with GDM who delivered between 2008 and 2012. Data were obtained from the analysis of the National Registry of GDM concerning these years (*n* = 8441) after permission from the Study Group of the Portuguese Society of Diabetology (*Sociedade Portuguesa de Diabetologia*), who coordinates this National Registry. Data are voluntarily and anonymously collected from clinical records of women with GDM, by multidisciplinary teams of Obstetrics and Diabetology, in 25 (of the 44) public Portuguese health institutions. These data sets are aggregated and validated by the Study Group of the Portuguese Society of Diabetology, according to the data provided by Portuguese Directorate-General of Health (*Direção Geral de Saúde*). Subsequently, the data sets are blinded relatively to the patients and hospitals’ identifications in order to unable investigators to identify the subjects and to maintain anonymity. All clinical investigations were conducted according to the principles expressed in the Declaration of Helsinki.

The GDM diagnosis was established using the criteria recommended by the Portuguese Directorate-General of Health. Until February 2011, the recommended GDM diagnosis was based on Carpenter and Coustan’s criteria [21]. After February 2011, the recommended diagnosis has been based on the International Association of Diabetes Pregnancy Study Groups’ criteria [22]. Each woman received detailed education on diet, exercise and appropriate glycemic targets (through self-directed glucose monitoring). Women were reviewed periodically depending on the stage of pregnancy. At each visit weight was measured and an assessment of previous blood glucose readings were made (insulin was started if they were outside the following ranges on more than 10 % of the measures: fasting glucose > 90 mg/dL or a 1 h postprandial reading of > 120 mg/dL).

The inclusion criteria include prepregnancy obesity (BMI ≥ 30 kg/m^2^). Prepregnancy BMI was calculated from self-reported prepregnancy weight and height. Women with missing GWG values and women with twin pregnancy were excluded.

GWG for the entire pregnancy was calculated and considering the 2009 IOM recommendations for obese women, we subsequently divided the assessed group into three categories: women who gained below (<5 kg), within (5–9Kg) and above (>9 kg) the IOM limits.

Neonates with a birthweight above the 90th percentile for gestational age were considered LGA, while those with a birthweight below the 10th percentile for gestational age were considered SGA [23]. Neonates with a birthweight > 4000 g were considered macrosomic. Gestational age was determined at the booking visit using obstetric ultrasound.

The analyzed data included biometric and demographic parameters, obstetric history, gestational age at diagnosis, need for insulin therapy, GWG, obstetric complications (such as preterm labor and cesarean section) and neonatal outcomes (including gestational age at delivery, birth weight, APGAR score, morbidities and admission to the intensive care unit).

Data were analyzed using STATA version 13.1. GWG was modeled as continuous and categorical variables (below, within and above IOM limits) to explain neonatal and maternal outcomes. We compared the obstetric, maternal and neonatal outcomes on the three categories (below, within and above IOM limits) using *χ*2 test to assess the differences in proportions and ANOVA to compare the means of continuous variables. Multivariate analyses were performed and adjusted odds ratio (aOR) were calculated using stepwise backward logistic regression models, adjusted for the following covariates: age, parity, prepregnancy BMI, use of insulin, gestational age at delivery, and birthweight (when appropriated). Data are expressed as proportions, means (±SD of the mean), adjusted odds ratios (aORs), and 95 % confidence intervals (CI). Statistical significance was accepted when the 95 % CI did not contain one (regression analyses). Significance was considered for *p* < 0,05.

## Results

The total cohort of the National Registry of GDM included 26 % (*n* = 2007) obese women (in a total of 7703 with registered prepregnancy BMI). After further exclusion of women with missing GWG values and with twin pregnancy, 1806 women met the eligibility criteria.

Only 35,1 % of obese women with GDM (*n* = 634) achieved the recommended GWG; 27,8 % (*n* = 502) gained below and 37,1 % (*n* = 670) gained above the IOM recommendations.

Table 1 summarizes, per category of GWG, the data on their demographics, rates of women on insulin therapy, delivery and neonatal outcomes.

**Table 1:** Characteristics of the study group (obese women with GDM)

		Total study group (*n* = 1806)	GWG below IOM limits (<5 kg) (*n* = 502)	GWG within IOM limits (5–9 kg) (*n* = 634)	GWG above IOM limits (>9 kg) (*n* = 670)
Age (years)		33.1 ± 5.0	33.4 ± 4.8	33.5 ± 4.9	32.5 ± 5.2**
Parity	= 0	38.4 % (*n* = 694)	38.1 % (*n* = 191)	37.5 % (*n* = 238)	39,6 % (*n* = 265)
	≥ 1	61.6 % (*n* = 1112)	61.9 %(*n* = 311)	62.5 % (*n* = 296)	60,4 % (*n* = 405)
Prepregnancy BMI (kg/m^2^)		34.7 ± 4.2	35.5 ± 4.5**	34.7 ± 4.1	34.1 ± 3.9
GWG (total) (kg)		8.1 ± 6.3	1.2 ± 3.2**	6.9 ± 1.3	14.3 ± 4.7**
Insulin therapy		47.2 % (*n* = 853)	50.2 % (*n* = 252)	45.9 % (*n* = 291)	46.3 % (*n* = 310)
Delivery (weeks)		38.4 ± 1.6	38.3 ± 1.8	38.3 ± 1.6	38.4 ± 1.4
Preterm labour		6.8 % (*n* = 123)	7.0 % (*n* = 35)	7.7 % (*n* = 49)	5.8 % (*n* = 39)
Delivery					
Normal delivery		40.5 % (*n* = 715)	47.8 % (*n* = 231)*	39.7 % (*n* = 247)	36.0 % (*n* = 237)
Instrumental delivery		11.4 % (*n* = 201)	11.6 % (*n* = 56)	11.4 % (*n* = 71)	11.2 % (*n* = 74)
Cesarean section		48.1 % (*n* = 849)	40.6 % (*n* = 196)*	48.9 % (*n* = *n* = 305)	52.8 % (*n* = 348)
Neonatal birthweight (g)		3312 ± 560	3169 ± 537**	3284 ± 560	3442 ± 548**
LGA (>p90)		15.8 % (*n* = 274)	9.1 % (*n* = 43)	14.2 % (*n* = 87)	22.3 % (*n* = 144)**
Macrosomic		9.0 % (*n* = 157)	4.6 % (*n* = 22)	8.2 % (*n* = 51)	12.8 % (*n* = 84)*
SGA (<p10)		4.9 % (*n* = 85)	7.8 % (*n* = 37)*	4.6 % (*n* = 28)	3.1 % (*n* = 20)
Neonatal morbidities		19.4 % (*n* = 295)	18.4 % (*n* = 73)	18.4 % (*n* = 98)	21.1 % (*n* = 124)
Admission to the intensive unit care		3.1 % (*n* = 48)	3.9 % (*n* = 16)	2.9 % (*n* = 16)	2.7 % (*n* = 16)

Data are expressed as mean ± SD and percentage and number of patients (in parentheses) of the total group

*p < 0.05 **p < 0.01 (both GWG below and GWG above IOM limits were compared with GWG within IOM limits)

Pregnant women who gained below the IOM limits had a higher prepregnancy BMI than the rest of the study population (*p* < 0,01).

The mean overall weight gain was 8,1 ± 6,3 kg. However, 4,6 % (*n* = 84) of obese women with GDM lost weight (−4,3 ± 3,7 kg).

Gestational age at delivery and the rates of preterm delivery (before 37 weeks) were similar in the three weight-gain groups (*p* > 0.05).

In obese women with GDM, the cesarean rate was significantly higher (48,1 %) than in total women of the National Registry of GDM (including all BMI’s categories) (37,2 %) (*p* < 0,01). Nevertheless, in obese women with GDM: when comparing with adequate GWG, rates of cesarean section were significantly lower in the group with GWG below IOM limits (40,6 %; *p* < 0,05), and even lower in the subgroup with gestational weight loss (27,2 %; *p* < 0,01).

Regarding neonatal birthweight, there was a positive correlation between GWG and neonatal birthweight (*r* = 0,225; *p* < 0,001). When compared with adequate GWG, birthweight was significantly lower in the group with insufficient GWG (*p* < 0,01) and significantly higher in the group with excessive GWG (*p* < 0,01).

LGA and macrosomic neonates were more common in the group with greater GWG (*p* < 0,01 and *p* < 0,05, respectively).

SGA neonates were more common in the group with GWG below IOM limits (7,8 %; *p* < 0,05); however, rates were similar between the subgroup with GWG between 0 and 5 kg and the subgroup with gestational weight loss (7,9 % versus 7,4 %; *p* > 0,05).

No differences in neonatal morbidities were found between the three weight-gain groups (*p* > 0,05).

Using multivariable analysis, GWG below IOM limits was associated with lower odds for cesarean section, even adjusting for birthweight [aOR 0,67 (0,54–0,85); *p* < 0,001]. Furthermore, it was associated with lower odds for LGA [aOR 0,39 (0,28–0,57); *p* < 0,001] and of macrosomia [aOR 0,34 (0,21–0,55); *p* < 0,001] (Table 2).

**Table 2:** Multivariable analysis of adverse outcomes associated with GWG (below versus above IOM limits) in obese women with GDM

Adverse outcomes	GWG below IOM limits (<5 kg)		GWG above IOM limits (>9 kg)	
	aOR (95 % CI)	*p* value	aOR (95 % CI)	*p* value
Cesarean section^a^	0.67 (0.54–0.85)	0.001	1.31 (1.07–1.61)	0.009
LGA^b^	0.39 (0.28–0.57)	<0.001	2.32 (1.76–3.04)	<0.001
Macrosomia^b^	0.34 (0.21–0.55)	<0.001	2.39 (1.68–3.38)	<0.001
SGA^b^	2.14 (1.36–3.35)	0.001	0.53 (0.31–0.88)	0.015
Low Apgar score^a^	0.14 (0.01–1.33)	>0.05	4.79 (1.19–19.21)	0.027
Admission to the intensive unit care^a^	1.38 (0.71–2.68)	>0.05	0.81 (0.42–1.54)	>0.05

^a^Adjusted for: age, parity, prepregnancy BMI, use of insulin, gestational age at delivery and birthweight (except for the aORs of the outcomes LGA, macrosomia and SGA)

^b^Adjusted for: age, parity, prepregnancy BMI, use of insulin, gestational age at delivery

On the other hand, GWG above IOM limits, even adjusting for birthweight, was associated with higher odds for cesarean section [aOR 1,31 (1,07–1,61); p 0,009] and of low Apgar score [aOR 4,79 (1,19–19,21); p 0,027]. It was also associated with higher odds for LGA [aOR 2,32 (1,76–3,04); *p* < 0,001] and of macrosomia [aOR2,39 (1,68–3,38); *p* < 0,001] (Table 2).

## Discussion

GDM, obesity and excessive GWG represent, individually and additively, high-risk conditions associated with adverse maternal and neonatal outcomes [2, 4, 5, 10, 24].

Women with excessive GWG were predominantly younger. As mentioned in a recent study [18], these data suggest that older women may be more likely to comply with lifestyle recommendations during pregnancy.

Overall, despite a regular multidisciplinary intervention program, more than a third of women with obesity and GDM gained excessive weight during pregnancy.

The rate of insulin treatment was similar in all three weight-gain groups, which suggests that glycemic control was similarly reached during the pregnancies.

In this high-risk group, we realized that GWG above IOM limits is significantly associated with an additive risk for LGA and macrosomia. These data are consistent with other authors who have shown similar effects of excessive GWG on birthweight for mixed populations of pregnant women [10, 12–14, 24] and specifically for women with GDM [18, 19]. LGA and macrosomic newborns had been associated with adverse short-term outcomes, such as an increased risk of instrumental vaginal delivery, emergency cesarean section, shoulder dystocia, fetal birth injury, postpartum hemorrhage, Apgar score <4 and admission to the intensive care unit and adverse long-term outcomes like childhood obesity, metabolic syndrome and cancer [4, 5, 7].

Additionally, we demonstrate that excessive GWG is significantly associated with an additional risk of cesarean section and low Apgar scores (5 min Apgar <7), even after adjusting for other factors including birthweight. A higher rate of cesarean section associated with excessive GWG has already been described in the general population [9, 12, 24], obese women [14, 15] and obese women with GDM [19]. This increased risk of cesarean section in obese women with GDM must be avoided because, not only does it pose immediate operative risks but it is also associated with post-operative complications such as wound infection or dehiscence, excessive blood loss, deep venous thrombosis and postpartum endometritis [4].

Thus, preventing excessive GWG is imperative. Considering the inherent difficulties of this high-risk group, preventing excessive GWG may be more feasible than prevention of obesity and GDM, as it is monitored during pregnancy.

Reduced GWG (<5 kg), in obese women with GDM, is significantly associated with a decreased risk of LGA and macrosomia, when adjusted for other relevant factors. Although the risk of SGA is increased in this group, using adequate percentiles birthweight curves to our population (published in 2011, by Pedreira et al. [23]), we verify that this risk is equivalent to the risk of SGA in low-risk pregnancies.

In addition, GWG below IOM recommendations, especially with weight loss, leads to a marked reduction in cesarean section rates and in all its possible associated complications.

These data support some previous studies which propose that reduced GWG (below IOM thresholds) leads to better maternal and neonatal outcomes in obese women [14, 25] as well as obese women with GDM [19, 20].

This study has limitations and among them is that it was not nationwide (it covered 25 of 44 units) and prepregnancy BMI was measured using self-reported information (prepregnancy weight and height), which may have been erroneous. However, there is evidence that self-reported pregnancy data tend to be accurate [26]. Also, 8.7 % (*n* = 738) of the pregnant women with GDM were excluded from the original cohort for missing prepregnancy BMI. In addition, GDM official recommendations for diagnosis and recommendations for GWG changed during the study period (respectively, 2011 and 2009) which may have affected some of our outcomes.

Importantly, this study also has strengths: the sample is large and it was controlled for potential confounders. In addition, this data is potentially relevant for public health measures because it could address some unresolved issues including the influence of GWG on maternal and neonatal outcomes of obese pregnant women with GDM.

## Conclusion

In obese women with GDM, a reduced GWG (<5 kg) is associated with better obstetric and neonatal outcomes than an excessive or even an adequate GWG (the ideal GWG for these women is less than the ideal for non-diabetic obese women). Thus, specific recommendations for obese women with GDM should be created because GWG could be a modifiable risk factor for adverse obstetric outcomes. Instructing women about appropriate GWG and implementing effective strategies (like diet adjustment and increased physical activity) to obtain the minimum GWG could help optimize maternal and perinatal outcomes.

## Abbreviations

GDM: Gestational diabetes mellitus
BMI: Body mass index
LGA: Large for gestational age
GWG: Gestational weight gain
SGA: Small for gestational age
IOM: Institute of medicine
aORs: Adjusted odds ratios
CI: Confidence intervals
